# Acute Contained Ruptured Aortic Aneurysm Presenting as Left Vocal Fold Immobility

**DOI:** 10.1155/2015/219090

**Published:** 2015-01-11

**Authors:** Sharon H. Gnagi, Brittany E. Howard, Joseph M. Hoxworth, David G. Lott

**Affiliations:** ^1^Department of Otolaryngology-Head and Neck Surgery, Mayo Clinic Arizona, 5777 East Mayo Boulevard, Phoenix, AZ 85054, USA; ^2^Department of Radiology, Mayo Clinic Arizona, 5777 East Mayo Boulevard, Phoenix, AZ 85054, USA

## Abstract

*Objective*. To recognize intrathoracic abnormalities, including expansion or rupture of aortic aneurysms, as a source of acute onset vocal fold immobility. *Methods*. A case report and review of the literature. *Results*. An 85-year-old female with prior history of an aortic aneurysm presented to a tertiary care facility with sudden onset hoarseness. On laryngoscopy, the left vocal fold was immobile in the paramedian position. A CT scan obtained that day revealed a new, large hematoma surrounding the upper descending aortic stent graft consistent with an acute contained ruptured aortic aneurysm. She was referred to the emergency department for evaluation and treatment by vascular surgery. She was counseled regarding surgical options and ultimately decided not to pursue further treatment. Her vocal fold immobility was subsequently treated via office-based injection medialization two weeks after presentation and again 5 months after the initial injection which dramatically improved her voice. Follow-up CT scan at 8 months demonstrated a reduction of the hematoma. The left vocal cord remains immobile to date. *Conclusion*. Ortner's syndrome, or cardiovocal syndrome, is hoarseness secondary to left recurrent laryngeal nerve palsy caused by cardiovascular pathology. It is a rare condition and, while typically presenting gradually, may also present with acute symptomatology.

## 1. Introduction

Ortner's syndrome, or cardiovocal syndrome, is hoarseness secondary to recurrent laryngeal nerve palsy caused by cardiovascular pathology. It is a rare condition and may present in an acute fashion representing an underlying acute cardiovascular event. We present a case of acute contained aortic aneurysm rupture presenting with sudden onset hoarseness. This study was exempted by the Institutional Review Board at Mayo Clinic.

## 2. Case Description

**Figure 1:**
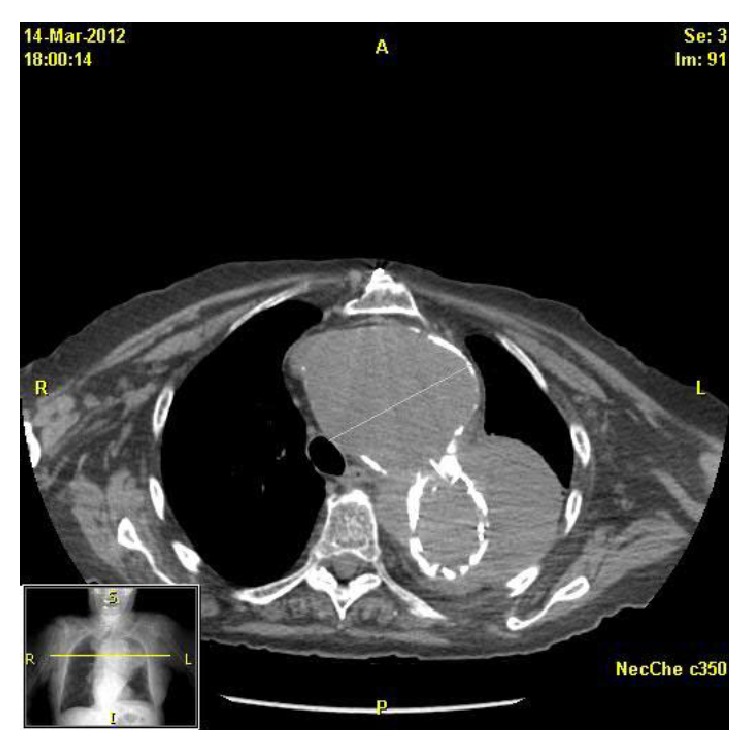
Large acute hematoma surrounding the proximal descending thoracic aorta with insinuation of the hematoma into the aortopulmonary window, concerning in appearance for contained rupture.

An 85-year-old woman with past medical history significant for an aortic aneurysm status after repair presented to a tertiary care laryngology clinic with a history of sudden onset hoarseness. She denied any other acute symptom changes including dysphagia, odynophagia, chest, neck, or abdominal pain, cough, and neurologic changes. On laryngoscopy, the left true vocal fold was nonmobile and in a paramedian position. Given her aneurysm history, a CT scan was obtained that day. It revealed a new, large acute 4.6 cm hematoma surrounding the upper descending aortic stent graft consistent with an acute contained ruptured aortic aneurysm (Figure 1). She was subsequently referred to the emergency department for further evaluation and treatment by the vascular surgery team. On presentation, her vital signs were stable with a blood pressure of 122/60 and a heart rate of 86. She was afebrile, with normal respiratory rate and normal oxygen saturation on room air. On further examination, she did have a murmur consistent with aortic regurgitation with no radiation to the carotids. There was no carotid bruit or jugular venous distention. She had symmetric 2+ pulses in bilateral upper and lower extremities. The remainder of her examination was normal. After discussion with both vascular and cardiovascular surgeons, she was counseled regarding surgical options and ultimately decided not to pursue further treatment. Secondary to her poor surgical candidacy, her vocal fold immobility was subsequently treated via office-based injection medialization. She was injected two weeks after presentation and again 5 months after the initial injection which dramatically improved her voice. Follow-up CT scan at 8 months after diagnosis demonstrated a slight decrease in the size of the hematoma with stabile size of the aneurysm itself. The left vocal fold remains immobile to date.

## 3. Discussion

Ortner originally described recurrent laryngeal nerve palsy in association with severe mitral stenosis causing left atrial enlargement in 1897 [1]. Since that time, cardiovascular pathology associated with vocal fold immobility has been known as Ortner's syndrome or cardiovocal syndrome. Ortner's syndrome commonly affects the left recurrent laryngeal nerve as it courses deeper in the mediastinum through the aortopulmonary window, behind the ligamentum arteriosum, and around the aortic arch. Ortner's syndrome has been seen with cardiac enlargement due to various underlying pathologies, cardiac tumors, aortic aneurysm with or without rupture, and aortic dissection among other etiologies.

Pain is the most common coexisting symptom and may be present in the chest, back, or abdomen. The pain is abrupt in onset, severe in intensity, and may have a ripping, tearing, sharp, or stabbing quality. While 90% of aortic dissections present with pain, 10% may be painless [2]. In these patients, vocal fold immobility may be one of the first and only symptoms leading the patient to be evaluated by a physician. Vocal fold paresis may also be associated with imminent rupture [3], a typically fatal event with 40% of patients dying immediately, 1% per hour dying thereafter, and between 5 and 20% dying during or shortly after surgery [4]. Other coexisting historical findings concerning rupture or dissection are seen in Table 1 [4]. Although not seen in this case, several recent reports have noted acute or progressive onset of dysphagia [5, 6] or cough [7] with hoarseness at presentation. High risk physical exam findings may include a new aortic insufficiency murmur, hypotension or shock state, pulse deficits, focal neurologic deficit, or systolic blood pressure limb differential greater than 20 mmHg [4]. Secondary to the life-threatening nature of these conditions, early recognition and treatment is a priority. Therefore, the otolaryngologist should be aware of these risk factors and subsequently perform appropriate imaging in a timely manner.

Computed tomography of the neck and chest is recommended to evaluate the course of the recurrent laryngeal nerve in the setting of acute vocal fold immobility [8]. This will detect common pathologies associated with vocal fold immobility such as malignancy, cardiac enlargement, and mediastinal lymphadenopathy, in addition to acute cardiovascular events such as aortic dissection and aneurysm rupture. In most settings, imaging may be performed on a routine basis; however, high risk patients such as those with pain, physical findings as described above, previous history of aneurysm, and those with features in Table 1 may warrant a more urgent imaging evaluation to avoid catastrophic complications [4].

As in our case, many patients with cardiovocal syndrome secondary to aortic aneurysms are poor surgical candidates for correction of their vocal fold immobility and may benefit from either office-based transoral injection medialization or a medialization thyroplasty under local anesthesia [9]. While Ortner's syndrome secondary to aortic dissection or aneurysm rupture is rare, there appear to be an increasing number of published case reports regarding aortic aneurysms presenting with hoarseness in the last decade [3, 5–7, 9–12]. This may reflect improved diagnosis and survival secondary to medical and surgical advances. Moving forward, this further supports that otolaryngologists must be aware of this and include it in the differential diagnosis for vocal fold immobility.

## 4. Conclusion

Vocal fold immobility may be the initial and only sign of serious cardiovascular pathology. Although rare, aortic dissection or aneurysm rupture may present as acute vocal fold immobility without coexisting symptoms. Given the exceedingly high mortality of these conditions, otolaryngologists must observe a high degree of suspicion in patients presenting with acute vocal fold immobility and high risk features. The authors propose that these patients be promptly evaluated with imaging to rule out an acute cardiovascular event.

## Figures and Tables

**Table 1:** High risk features for aneurysm rupture/dissection [4].

*Historical features* (i) Pain (back, chest, and epigastrium) (ii) Syncopal episode (iii) Trauma (deceleration or torsional injury) (iv) History of extreme exertion or emotional distress just prior to onset (v) Pregnancy (vi) Cocaine or other stimulants use *Past medical history* (i) Known thoracic aortic aneurysm (ii) Known aortic valve disease (iii) Hypertension (especially uncontrolled) (iv) Bicuspid aortic valve (v) Coarctation of the aorta (vi) Aberrant right subclavian artery (vii) Right aortic arch (viii) Pheochromocytoma (ix) Chronic corticosteroid or immunosuppression agent use (x) Human immunodeficiency virus (xi) Infections of aortic wall (bacteria, fungi, tuberculosis, and syphilis)	*Past surgical history* (i) Aortic valve replacement (ii) Recent aortic manipulation (surgical or catheter-based) *Familial history of thoracic aortic disease* (i) Familial thoracic aortic aneurysms and dissection syndrome (ii) Bicuspid aortic valve *Predisposing genetic syndromes* (i) Marfan syndrome (ii) Ehlers-Danlos syndrome (iii) Turner syndrome (iv) Loeys-Dietz syndrome (v) Polycystic kidney disease (vi) Noonan syndrome (vii) Alagille syndrome (viii) Congenital contractural arachnodactyly (ix) Beals syndrome *Predisposing inflammatory conditions* (i) Giant cell arteritis (ii) Takayasu arteritis (iii) Behçet disease (iv) Ankylosing spondylitis
